# SARS-CoV-2 surveillance in Italy through phylogenomic inferences based on Hamming distances derived from pan-SNPs, -MNPs and -InDels

**DOI:** 10.1186/s12864-021-08112-0

**Published:** 2021-10-30

**Authors:** Adriano Di Pasquale, Nicolas Radomski, Iolanda Mangone, Paolo Calistri, Alessio Lorusso, Cesare Cammà

**Affiliations:** National Reference Centre (NRC) for Whole Genome Sequencing of microbial pathogens: data-base and bioinformatics analysis (GENPAT), Istituto Zooprofilattico Sperimentale dell’Abruzzo e del Molise “Giuseppe Caporale” (IZSAM), via Campo Boario, 64100 Teramo, TE Italy

**Keywords:** SARS-CoV-2, Surveillance, Italy, Abruzzo, Hamming distances, Minimum spanning tree

## Abstract

**Background:**

Faced with the ongoing global pandemic of coronavirus disease, the ‘National Reference Centre for Whole Genome Sequencing of microbial pathogens: database and bioinformatic analysis’ (GENPAT) formally established at the ‘Istituto Zooprofilattico Sperimentale dell’Abruzzo e del Molise’ (IZSAM) in Teramo (Italy) is in charge of the SARS-CoV-2 surveillance at the genomic scale. In a context of SARS-CoV-2 surveillance requiring correct and fast assessment of epidemiological clusters from substantial amount of samples, the present study proposes an analytical workflow for identifying accurately the PANGO lineages of SARS-CoV-2 samples and building of discriminant minimum spanning trees (MST) bypassing the usual time consuming phylogenomic inferences based on multiple sequence alignment (MSA) and substitution model.

**Results:**

GENPAT constituted two collections of SARS-CoV-2 samples. The first collection consisted of SARS-CoV-2 positive swabs collected by IZSAM from the Abruzzo region (Italy), then sequenced by next generation sequencing (NGS) and analyzed in GENPAT (*n* = 1592), while the second collection included samples from several Italian provinces and retrieved from the reference Global Initiative on Sharing All Influenza Data (GISAID) (*n* = 17,201). The main results of the present work showed that (i) GENPAT and GISAID detected the same PANGO lineages, (ii) the PANGO lineages B.1.177 (i.e. historical in Italy) and B.1.1.7 (i.e. ‘UK variant’) are major concerns today in several Italian provinces, and the new MST-based method (iii) clusters most of the PANGO lineages together, (iv) with a higher dicriminatory power than PANGO lineages, (v) and faster that the usual phylogenomic methods based on MSA and substitution model.

**Conclusions:**

The genome sequencing efforts of Italian provinces, combined with a structured national system of NGS data management, provided support for surveillance SARS-CoV-2 in Italy. We propose to build phylogenomic trees of SARS-CoV-2 variants through an accurate, discriminant and fast MST-based method avoiding the typical time consuming steps related to MSA and substitution model-based phylogenomic inference.

**Supplementary Information:**

The online version contains supplementary material available at 10.1186/s12864-021-08112-0.

## Introduction

The coronavirus disease 19 (COVID-19) responsible to the current pandemic is due to a novel coronavirus (CoV) named SARS-CoV-2 [1]. COVID-19 was firstly reported in humans during December 2019 in the city of Wuhan (Hubei Province, China) and the role of the Huanan seafood wholesale market in SARS-CoV-2 emergence is still uncertain [2–4]. At the date of the present study (May 2021), 222 countries were affected by the SARS-CoV-2 with 153,527,666 coronavirus cases, as well as 3,217,267 deaths, 680,364 daily new cases, and 9981 daily deaths [5]. With more than 1000 cases confirmed till the 1^st^ March 2020, Italy was one of the first European countries to face the SARS-CoV-2 burden [6]. At the national level, the Italian Civil Protection Department counted today 4,044,762 total cases, 121,177 deaths, 3,492,679 recovered people and 430,906 active cases in Italy [7]. COVID-19 is mainly a respiratory infection, with the most common symptoms comprising fever, dry cough, and shortness of breath [8]. About 20% of infected patients may develop severe disease, and a small percentage (5%) may become critically ill [8]. Patients with severe COVID-19 disease may develop pneumonia or acute respiratory distress syndrome (ARDS), which is often fatal [9] and requires mechanical ventilation and treatment from intensive care unit [8].

CoVs harbor enveloped single-stranded RNA genomes with high plasticity [10] induced by a high-frequency RNA recombination [2, 3]. New genotypes have emerged from homologous RNA recombination, and novel genes have been acquired through heterogeneous RNA recombination with non-coronaviral donor RNAs [11]. SARS-CoV-2 is paradigmatic of these evolutionary mechanisms as there is compelling evidence that it emerged through recombination of SARS-related coronaviruses (SARSr-CoVs) as it was suggested for SARS-CoV-1 [2, 3, 12, 13]. Besides recombination events, the point mutations from replication errors drive also the SARS-CoV-2 evolution (i.e. single nucleotide polymorphisms (SNPs), multi-nucleotide polymorphisms (MNPs) and small insertions/deletions (InDels)) [14]. The likely SNP-based mutation rate of the SARS-CoV-2 (~ 10^− 6^ nt^− 1^ cycle^− 1^) is low compared to influenza virus (~ 3 × 10^− 5^ nt^− 1^ cycle^− 1^) or other RNA viruses [15]. In fact, the SARS-CoV-2 is able to repair part of duplication errors induced by the RNA-dependent RNA polymerases (RdRp) [16]. However, a SARS-CoV-2 population in one milliliter of sputum (i.e. around 10^7^ RNAs/ml) with this likely mutation rate (~ 10^− 6^ nt^− 1^ cycle^− 1^) would harbor more than one mutation in every nucleotide [17], not mentioning that spreading over millions of individuals induces fast accumulation of mutations.

In addition to negative impacts of the SARS-CoV-2 on hospital workload [18], medical clinic organization [19], long-term health [20], small business [21], socio-economic system [22] and employment [23], the national health care systems have to face the need for thousands of laboratory tests per day [24]. The Veterinary Public Health Institutes, namely Istituti Zooprofilattici Sperimentali (IZS), perform the diagnosis of SARS-CoV-2 through testing nasopharyngeal swabs by RT-PCR on behalf of the Italian Ministry of Health [24]. In the face of the current COVID-19 crisis, the “National Reference Centre for Whole Genome Sequencing of microbial pathogens: database and bioinformatic analysis” (GENPAT) formally established at the IZS dell’Abruzzo e del Molise (IZSAM) in Teramo (G.U.R.I. 196, August 23, 2017), dedicates its developments to improve analytical workflows of SARS-CoV-2 sequences from routine surveillance activities.

Different international teams proposed analytical workflows to reconstruct SARS-CoV-2 genomes based on de novo assemblies [25, 26] and/or consensus sequences [27] from variant calling analysis [28–30] performed through mapping of reads [26, 28–31] against the reference genome Wuhan-Hu-1/2019. The resulted de novo assemblies and consensus sequences are commonly uploaded at the international level into the Global Initiative on Sharing All Influenza Data (GISAID) [32]. From the de novo assemblies or consensus sequences, the dedicated PANGOLIN tool performs the identification of SARS-CoV-2 lineages, so-called PANGO lineages [33], and has been adopted by the reference GISAID [32] because it allows sharing between laboratories of a common dynamic nomenclature of mutations associated with important functional evolution events [34]. Otherwise, these de novo assemblies and consensus sequences are usually aligned between each other through multiple sequence alignment (MSA) [28, 35–37] in order to perform substitution model-based phylogenomic inferences through maximum likelihood (ML) [28, 35] or Bayesian models [37]. The aligned de novo assemblies and consensus sequences can also be derived into variant calling format (i.e. VCF) [37]. Because the biological effects of variants (i.e. SNPs, MNPs and InDels, so-called genotypes in VCF files) are required for identifying mutations associated with important functional evolution events [34] and accordingly designing of SARS-CoV-2 vaccines [38], these VCF files or aligned sequences are typically input data of functional annotation of variants [28, 29, 37]. Even though de novo assembly [25, 39], mapping of reads [40–43] and variant calling analysis [44–46] are relatively fast processes, these SARS-CoV-2 workflows are currently limited by the time consuming steps aiming at performing MSA [47–52], then substitution model-based phylogenomic inferences [53–55]. In fact, the phylogenetic inferences based on MSA and substitution model can take many days or weeks depending of the available computing power, particularly when the dataset of samples includes several hundreds of genomes.

In the area of surveillance dedicated to bacteria including the genera *Enterococcus* [56], *Mycoplasma* [57], *Pseudomonas* [58], *Mycobacterium* [59], *Brucella* [60] and many others, coregenome and whole genome multi-locus sequence typing (cg/wgMLST) and corresponding schemes of alleles have been proposed to identify epidemiological relationships based on screening of alleles through several hundred or thousands of homologous genes, so-called loci [61]. In comparison with the so-called allele scheme, the combination of these MLST allele numbers from a single strain allows assignation of a MLST sequence type (ST) already shared between laboratories or a new one by default [62]. The output of cg/wgMLST methods from different analytical workflows (e.g. chewBBACA [63], SeqSphere^+^ [64], MLSTar [65], BIGSdb-Pasteur [66], Bionumerics [67]) are frequently used as input for a recent minimum spanning tree (MST) algorithm (“MSTree V2”) implemented in the workflow GrapeTree in order to visualize coregenome relationships among hundreds of thousands bacterial genomes [68]. Compared to the good practices aiming at building a phylogenomic tree based on MSA, a substitution model (i.e. JC69, K80, K81, F81, HKY85, T92, TN93, or GTR) and an inference approch (i.e. ML or Bayesian models) [69, 70], the construction of a MST with “MSTree V2” is theoretically faster because it implements a directional measure based on normalized asymmetric Hamming-like distances between pairs of STs assuming that one of the pair of STs is the ancestor of the other [68].

Considering that the SARS-CoV-2 surveillance needs an accurate, discriminant and fast assessment of epidemiological clusters from substantial amount of samples, the present study provides a variant calling analysis-based workflow, so-called GENPAT workflow, to accurately identify the PANGO lineages of SARS-CoV-2 samples in Italy and rapidly build highly discriminant MST bypassing the usual time consuming phylogenomic inferences based on multiple sequence alignment (MSA) and substitution model. More precisely, the present manuscript aims at answering the following questions:

- Question i: Is the GENPAT workflow able to identify PANGO lineages compared to the reference GISAID?
- Question ii: What do the sequencing effort in Italy and GENPAT workflow development in the Abruzzo region reveal about the PANGO lineages mainly circulating in Italian provinces?
- Question iii: Does the MST-based clustering match the reference PANGO lineages and/or Italian provinces?
- Question iv: What are the differences of discrimination power between the developed MST-based method and PANGO lineages?
- Question v: What are the differences of speed between the developed MST-based method and the usual phylogenomic inferences based on MSA and substitution model?

## Results

**Fig. 1:**
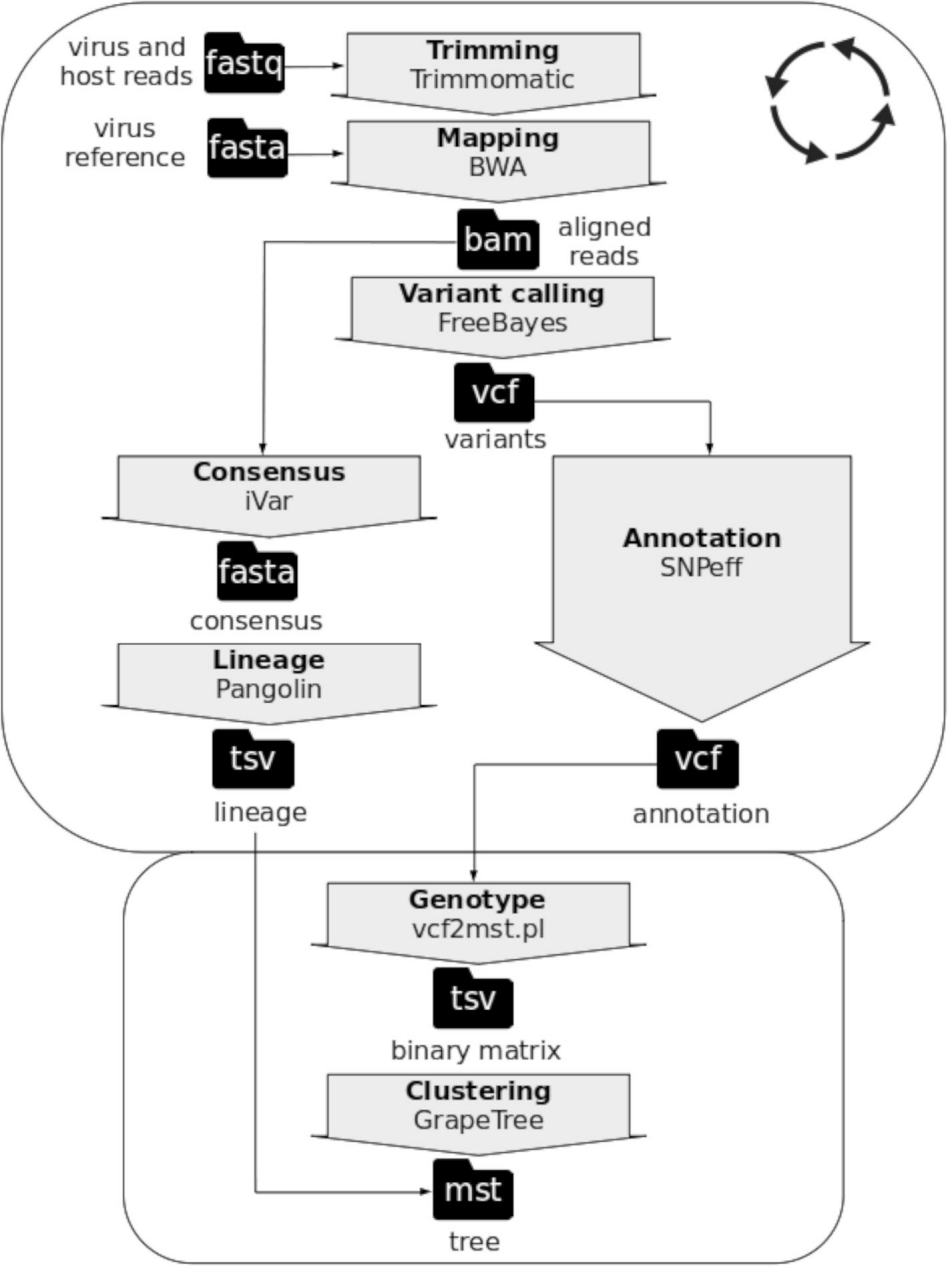
Sample dependent (rounded rectangle with circle of arrows) and dataset dependent (rounded rectangle without circle of arrows) steps of the workflow implemented in GENPAT to identify lineages of SARS-CoV-2 and build phylogenomic inference based on shotgun metagenomics paired-end read sequencing

**Fig. 2:**
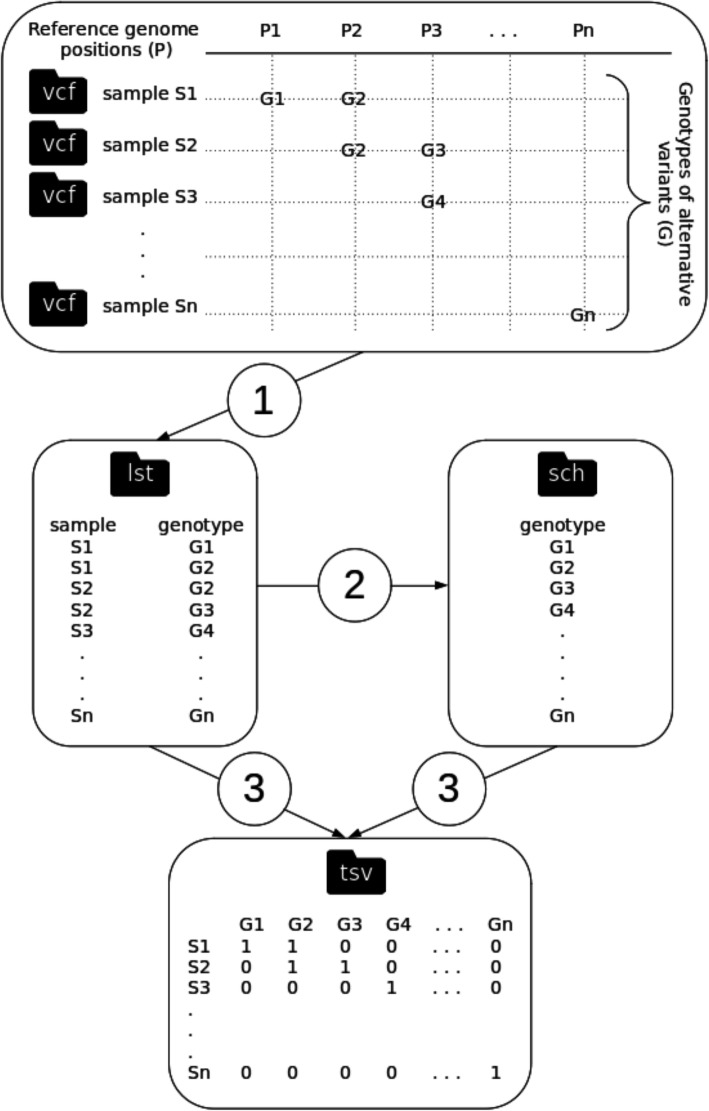
Algorithmic steps of the program “vcf2mst.pl” aiming to (1) derive functional annotations of variants (i.e. genotypes of pan-SNPs, -MNPs and -InDels) encoded in variant calling format (vcf) into lists of samples and genotypes (lst), (2) build a scheme of genotypes (sch) and (3) create a binary matrix of genotypes according to samples involved in reference genome based-variant calling analysis (tsv) for downstream inference of minimum spanning tree (MST)

The questions described above (i.e. questions i, ii, iii, iv and v) were assessed with the GENPAT workflow combining the identification of PANGO lineages based on variant calling analysis (Fig. 1) and MST-based phylogenomic inference (Figs. 1 and 2), as well as two collections of SARS-CoV-2 samples isolated until April 2021 in Italy from GENPAT (Additional file 1) and GISAID (Additional file 2).

### GENPAT workflow ability to identify PANGO lineages in comparison to the reference GISAID

The GENPAT collection corresponds to samples isolated by IZSAM in the Abruzzo region, then sequenced by NGS and analyzed in GENPAT, while the GISAID collection corresponds to samples isolated in Italy and analyses by the reference GISAID. Comparing the collections GENPAT (Additional file 1, *n* = 1592 samples) and GISAID (Additional file 2, *n* = 17,201 samples), 1550 common SARS-CoV-2 samples presented identical PANGO lineages. In response to question i, the GENPAT workflow (Fig. 1) is as precise as the reference GISAID to identify PANGO lineages (Additional files 1 and 2).

### Main PANGO lineages circulating in Italian provinces revealed by Italian genome sequencing activities and GENPAT workflow development

**Fig. 3:**
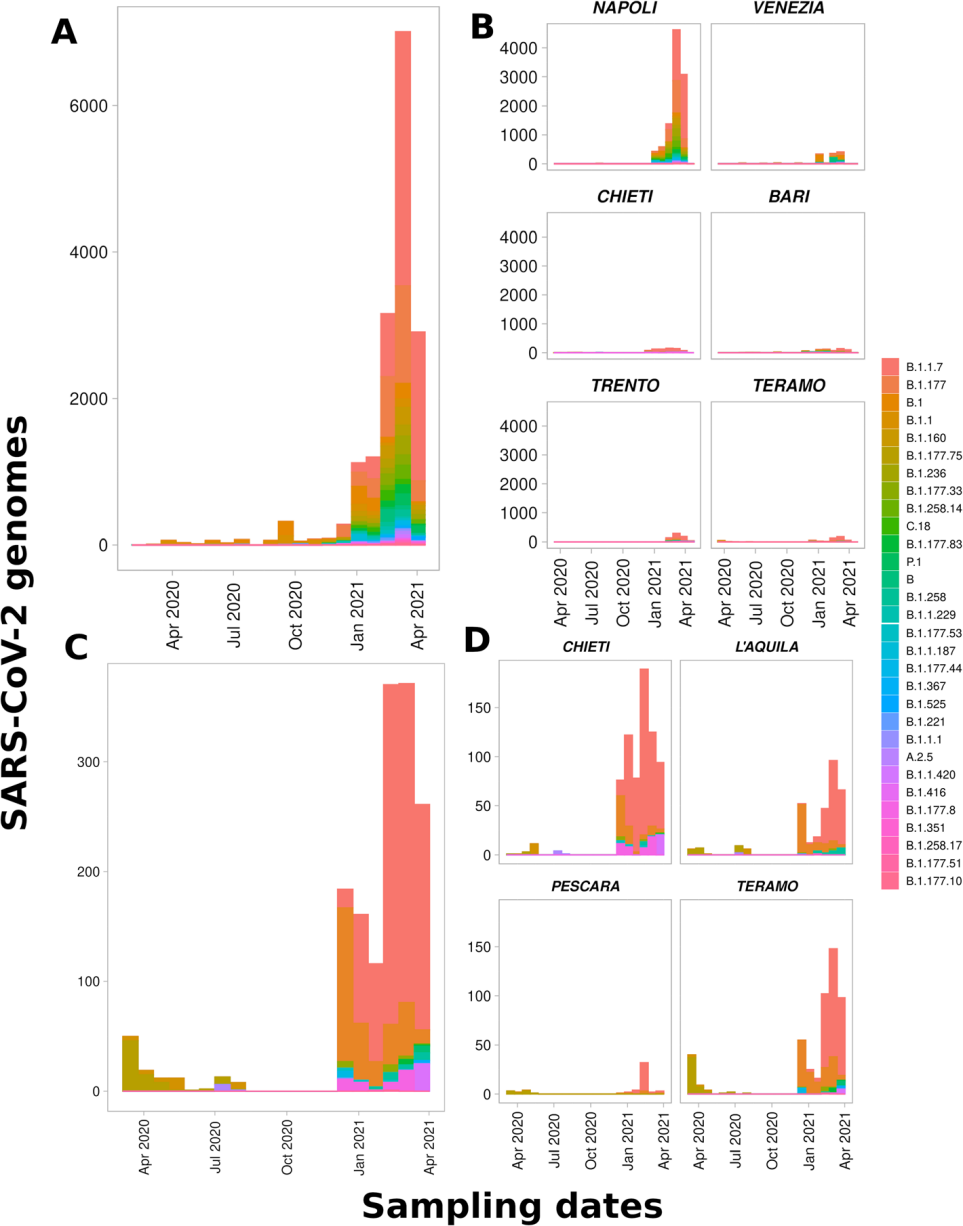
Distributions of the 30 most frequent SARS-CoV-2 PANGO lineages in Italy centralized in GISAID and represented at the national level (A, *n* = 16,529); among the 6 most frequent provinces in Italy including Napoli (*n* = 10,115), Venezia (*n* = 1214), Chieti (*n* = 705), Bari (*n* = 661), Trento (*n* = 632) and Teroma (*n* = 501) (B, *n* = 13,828); among samples from the Abruzzo region mainly shotgun sequenced and analyzed in GENPAT (C, *n* = 1580); and inside the provinces of the Abruzzo region including Chieti (*n* = 705), Teromo (*n* = 501), l’Aquila (*n* = 320) and Percara (*n* = 54) (D)

In view of the GISAID collection (Additional file 2, *n* = 17,201), the PANGO lineages B.1.1.7 (39%) and B.1.177 (17%) were the mostly identified in Italy (Fig. 3A), especially the province Napoli (24 and 12%) (*n* = 4184 and *n* = 2073) and, to a lesser extent, in the provinces of Venezia, Chieti, Bari, Trento and Teramo (Fig. 3B). With respect to the GISAID collection (Additional file 2), the lineages B.1.1.7 (39%) and B.1.177 (17%) were also the mostly detected in the Abruzzo region (Fig. 3C) and provinces of the Abruzzo region (Fig. 3D). Indeed, the PANGO lineages B.1.1.7 (62%) and B.1.177 (19%) were the mostly identified in the provinces of the Abruzzo region, namely Chieti (32 and 5%), L’Aquila (11 and 5%), Pescara (2% and 4‰) and Teramo (16 and 8%) (Table 1), among the SARS-CoV-2 samples from the GENPAT collection (Additional file 1, *n* = 1592). While the Napoli province produced the highest number of SARS-CoV-2 strain characterization in Italy (Fig. 3B, *n* = 10,372: 67%), the Chieti province presented the highest number of SARS-CoV-2 strains with an identified lineage in the Abruzzo region (Fig. 3B, *n* = 710: 45%). In response to question ii, the genome sequencing effort in Italy (i.e. GISAID collection) and GENPAT workflow developed in the Abruzzo region (i.e. GENPAT collection) revealed that PANGO lineages B.1.1.7 and B.1.177 have mainly circulating in Italy at the date of the present study.

**Table 1:** Distributions of PANGO lineages from SARS-CoV-2 samples retrieved in provinces of the Abruzzo region in Italy, then shotgun sequenced and analyzed by GENPAT until April 2021 (*n* = 1592)

Lineages	Provinces of the Abruzzo region			
	Chieti	L’Aquila	Pescara	Teramo
B.1	10	8	2	5
B.1.1	1	15	5	35
B.1.1.1	3	0	0	0
B.1.1.189	0	1	0	0
B.1.1.208	1	0	0	0
B.1.1.211	0	1	0	3
B.1.1.229	0	0	0	6
B.1.1.29	8	0	3	0
B.1.1.305	0	0	0	2
B.1.1.39	0	0	0	1
B.1.1.420	22	0	0	5
B.1.1.7	520	183	41	255
B.1.1.71	0	0	0	1
B.1.1.74	1	0	0	0
B.1.160	9	3	1	6
B.1.177	89	86	7	134
B.1.177.16	0	0	0	2
B.1.177.6	0	1	0	0
B.1.177.7	1	0	0	1
B.1.177.75	1	0	0	0
B.1.177.8	46	0	0	1
B.1.177.83	1	0	0	5
B.1.221	0	1	0	0
B.1.235	0	1	0	0
B.1.258	13	0	0	0
B.1.258.14	0	0	0	6
B.1.258.17	0	1	0	0
B.1.5	8	2	1	2
B.1.525	0	0	0	3
P.1	5	16	0	1

### Clustering by the MST-based method in comparison with the reference PANGO lineages

**Fig. 4:**
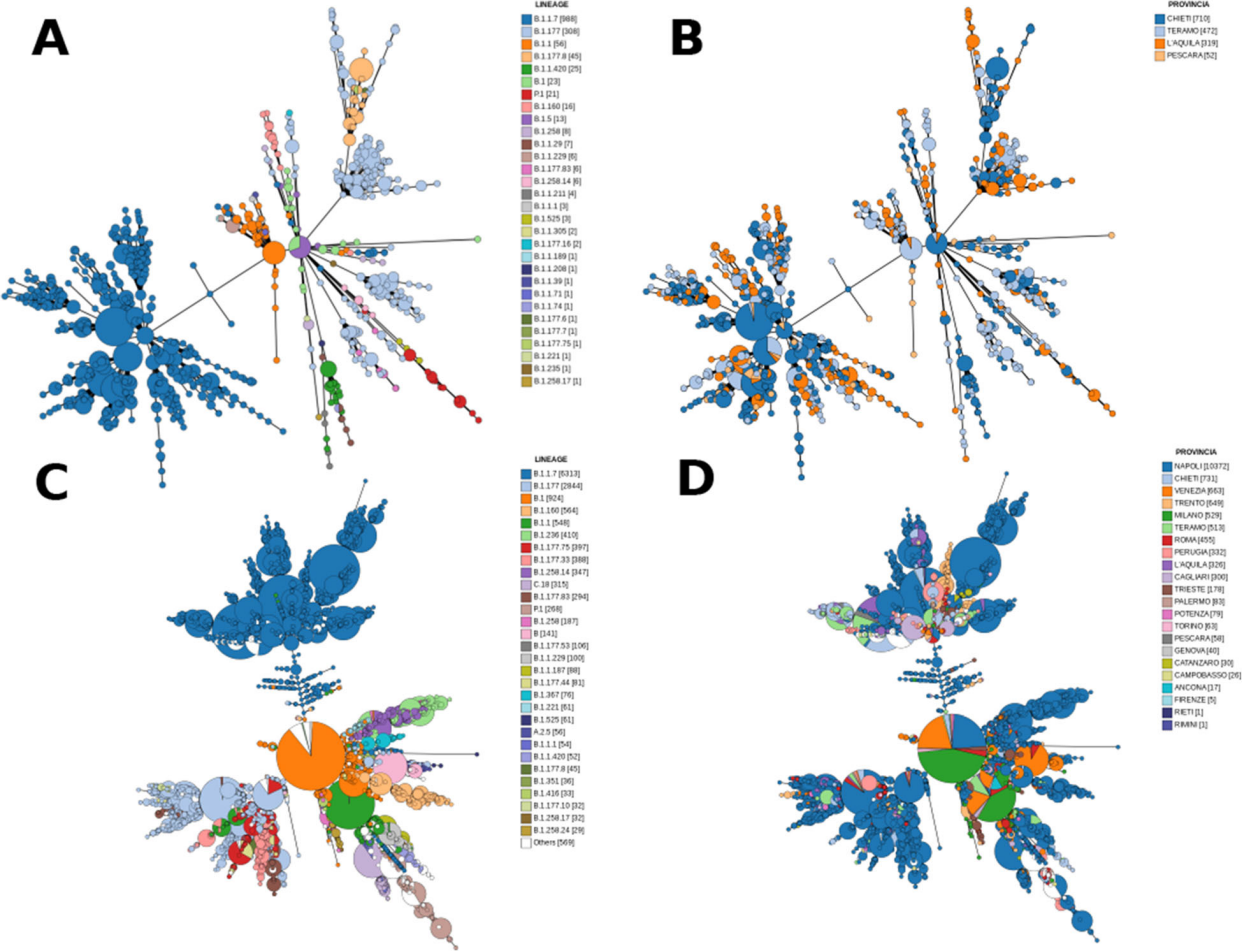
Pangenomic minimum spanning tree (MST) produced by the GENPAT workflow from SARS-CoV-2 samples isolated in provinces of the Abruzzo region and sequenced by GENPAT (A and B, *n* = 1553) or SARS-CoV-2 samples isolated in several Italian provinces and retrieved from GISAID (C and D, *n* = 15,451) with regard to lineages identified through PANGO dynamic nomenclature (A and C) or Italian provinces (B and D). The effective sample sizes are shown in square brackets

The GENPAT workflow provided MST-based clustering (Figs. 1 and 2) as exemplified by trees representing SARS-CoV-2 samples from the collections GENPAT (Fig. 4A and B, *n* = 1553) or GISAID (Fig. 4C and D, *n* = 15,451). For both collections GENPAT or GISAID including 30 and 176 PANGO lineages (Additional files 1 and 2), almost all the MST-based clusters matched with the reference PANGO lineages (Fig. 4A and C), but did not correspond specifically to Italian provinces (Fig. 4B and D). In reply to the question iii, the MST-based clustering implemented in the GENPAT workflow matched well the reference PANGO lineages without specific segregation of Italian provinces.

### Discrimination power of the MST-based method in comparison with the reference PANGO lineages

The PANGO lineages B.1.1.7 and B.1.177 mostly identified in the Abruzzo province (Fig. 4A: 63 and 19%) and Italy (Fig. 4C: 40 and 18%), were both represented by multiple MST-based clusters (Fig. 3A and C). These multiple MST-based clusters were also observed for other less common PANGO lineages, such like B.1.1, B.1.177.8, B.1.1.420, B.1, P.1 and B.1.160 (Fig. 3A and C). In response to the question iii, the discrimination power of the MST-based method is higher than the reference PANGO lineages.

### Speed of the MST-based method in comparison with the usual phylogenomic inferences based on MSA and substitution model

While MSA and substitution model-based phylogenomic inference would require several days to several weeks to reconstruct evolution history of several hundreds of genomes with a usual computing facility (i.e. server harboring 32 Go RAM and 32 core CPUs), GENPAT estimates that 30 s and 4 s were necessary to treat 1000 samples with the algorithms “vcf2mst.pl” and “MSTree V2”, respectively (Additional files 4 and 5). Concerning the question v, the MST-based method developed by GENPAT appears faster than the usual phylogenomic inferences based on MSA and substitution model.

## Discussions

The correct detection of lineages (i) of concern (ii), as well as the accurate (iii), discriminant (iv) and fast (v) MST-based inference, are all in line with the SARS-CoV-2 surveillance requirements.

### Accurate GENPAT identification of PANGO lineages

Due to exact match between PANGO lineages identified by GENPAT and the reference GISAID (Additional files 1 and 2, *n* = 1550), we recommend to identify SARS-CoV-2 lineages based on trimming, mapping, consensus building and lineage identification implemented in Trimmomatic [71], BWA [42], iVar [27] and PANGOLIN 2.0 [34], respectively (Fig. 1). Faced to the diversity of methods to identify SARS-CoV-2 variants based on variant calling analysis (i.e. SAMtools [28], Freebayes [29], GATK4 [30]) from mapping outcomes (i.e. Minimap [28], Minimap2 [29], BWA [30, 31], Bowtie2 [26]), we also encourage to pursue comparisons of these methods because the SARS-CoV-2 lineages are identified based on SNPs and InDels [34], while InDels are known to be more difficult to identify than SNPs [72].

### Most frequent PANGO lineages B.1.177 and B.1.1.7 in Italy

Numerous lineages of SARS-CoV-2 emerged since the beginning of pandemic and, at the time of the manuscript preparation, three of them are today considered as global variants of concern: B.1.1.7, B.1.351, and P.1 (i.e. B.1.1.248 renamed B.1.1.28.1) [34]. These PANGO lineages B.1.1.7, B.1.351 and P1 were first detected in United Kingdom (UK) [73], South Africa [74] and Brazil [75], respectively. These lineages B.1.1.7, B.1.351, and P.1 replaced previous circulating variants in their original countries and spread to other countries in Europe, the Americas, and Asia [76]. There is an inordinate amount of concern for these three lineages because of likely reinfections due to reduced cross-protective immunity [77–79] and potential involvement in vaccine efficacy [80, 81]. While the PANGO lineage B.1.177 identified frequently in Abruzzo (Table 1) and Italy (Additional file 3) corresponds to one of the main lineage identified at the beginning of the pandemic event in Italy [82], the other frequently isolated PANGO lineage B.1.1.7 corresponds to the variant of concern called “UK variant” [75, 83–86]. In addition, GENPAT did not identified many samples corresponding to variants of concern named “South Africa” (B.1.351) [75, 83, 87, 88], “Japan-Brazil” (B.1.1.248 reclassified to B.1.1.28.1 - alias P.1) [75, 83, 87–90], “Nigeria” (B.1.1.207) [75, 91], “Denmark” (Y453F, 69–70deltaHV) [92, 93], “UK-Nigeria” (B.1.525) [94], and “Indian” (B.1.617) [95], neither in the Abruzzo region (Fig. 3C, Fig. 4AB, Table 1 and Additional file 1), or Italy (Fig. 3AB, Fig. 4CD, Additional files 2 and 3).

### Clustering of the MST-based method in agreement with the PANGO lineages

To our knowledge, it is the first time that variant calling analysis [44–46] and MST-based method [68] are combined to infer phylogenomic history of SARS-CoV-2 samples. The adaptation of the MST-based method usually used after cg/wgMLST characterization of bacterial draft assemblies [56–60], to variant calling analysis widely used for SARS-CoV-2 investigation [28–30], allowed building of an efficient clustering workflow (Figs. 1 and 2), in almost complete agreement with the outcomes of the reference PANGO lineages [34]. In the foreseeable future, we would like to tag the MST clusters to provide a unified method to type SARS-CoV-2 lineages and build a phylogenomic inference at the same time.

### High discriminatory power of the MST-based method

The fact that the two main PANGO lineages in Italy (i.e. B.1.1.7 and B.1.177) are constituted of multiple MST clusters, emphases that the proposed method (Figs. 1 and 2) has a higher discriminatory power than PANGO for SARS-CoV-2 typing (Fig. 3B and D). Indeed, the proposed MST-based phylogenomic inference is able to manage together pan-SNPs, -MNPs and -InDels (i.e. core and accessory variants) with respect to the reference genome. The present MST-based method (Figs. 1 and 2) is also able to build MST only based on genotypes from functional annotations of variants identified in specific SARS-CoV-2 epitopes. This useful option of the algorithm “vcf2mst.pl” aims at providing graphical warnings related to SARS-CoV-2 mutations acquired in regions known to be involved in immune responses [96]. Another useful feature of the algorithm “vcf2mst.pl” is a capacity to manage nucleotide (i.e. GENPAT collection) or amino acid (i.e. GISAID collection) patterns for users who only have one type of data.

### Fast minimum spanning tree from function annotations of variants

In comparison to the time consuming steps (i.e. several days or weeks for thousands samples) aiming at performing MSA from de novo assemblies or consensus sequences [28, 31, 35, 36], as well as phylogenomic inferences based on substitution models [28, 35, 37], the Hamming distance-based method [68] developed in the present manuscript (Figs. 1 and 2) is very fast (i.e. tens of seconds to process thousands of samples). Even if this MST-based method does not root trees and does not take in account differences of evolution rates between lineages [68], this last allows fast graphical representation of SARS-CoV-2 spreading for surveillance requiring fast assessment of epidemiological clusters from substantial amount of samples (Fig. 4). In agreement with the World Health Organization (WHO), the rapid generation and sharing of virus genomic sequences will contribute to the understanding of transmission and the design of mitigation strategies [97]. Collaboration between public health bodies, data generators and analysts is essential to generate and use appropriately data for maximum public health benefit [97]. Concerning the research studies supporting that the SARS-CoV-2 emerged firstly from China in late 2019 [98–101] (i.e. firstly reported in December 2019 [4, 102, 103] with a plausible emergence between early October [104], or mid-October, and mid-November 2019 [105]), or from other countries at a similar period [106], or even earlier [107], the phylogenomic inference at a pangenomic scale based on MSA and substitution models [28, 35–37] remains the gold standard to confirm the geographical origin(s) of SARS-CoV-2 spreading, because our MST-based method does not root trees and does not integrate differences of evolution rates between lineages [68].

## Conclusion

The main results of the present developments showed that (i) GENPAT and GISAID detected the same PANGO lineages, (ii) the PANGO lineages B.1.177 (i.e. historical in Italy) and B.1.1.7 (i.e. “UK variant”) are major concerns today in several Italian provinces, and the new MST-based method (iii) clusters most of the PANGO lineages together, (iv) with a higher discriminatory power than the PANGO lineages, (v) and faster than the usual phylogenomic methods based on MSA and substitution model. The genome sequencing efforts of Italian provinces, combined to a structured national management of metagenomics data, provided an accurate and fast answer supporting the system of SARS-CoV-2 surveillance in Italy. In addition, the outcomes of the present consortium involved in SARS-CoV-2 surveillance in Italy emphasized that the data sharing through GISAID is of paramount importance for supporting the international SARS-CoV-2 tracking.

## Material and methods

A workflow was implemented in GENPAT during 2021 to identify SARS-CoV-2 lineages and build accurate, discriminant and fast phylogenomic inferences from several thousands of samples isolated in Italy based on shotgun metagenomics paired-end read sequencing (Fig. 1). In the present study, the adjective ‘discriminant’ refers to a high discriminatory power and the term ‘discriminatory power’ is defined as the ability of a molecular typing method to distinguish between two or more groups being assessed [108].

### Collections of SARS-CoV-2 samples

Two collections of SARS-CoV-2 samples were established (i.e. metadata, lineages and functional annotation of variants). The first collection includes 1592 SARS-CoV-2 positive swab samples detected by IZSAM until April 2021 in the Abruzzo region (Italy), then sequenced by NGS and analyzed in GENPAT. Sequences were then systematically submitted by GENPAT to GISAID (https://www.gisaid.org/) [32]. The second collection harbors 17,201 samples isolated from different Italian regions and downloaded by GENPAT from GISAID in April 2021 [32]. While samples from the first collection were treated through the whole GENPAT workflow, those from the second collection were treated through the dataset dependent part of this workflow based on information retrieved from GISAID (Fig. 1).

### SARS-CoV-2 detection and genome sequencing

Concerning the samples from the first collection, acquisition of sequencing data implied successively sampling (oropharyngeal swab transport medium or bronchoalveolar lavage), virus inactivation (PrimeStore® MTM, in BSL3 biocontainment laboratory), nucleic acid purification (MagMaxTM CORE from Thermofisher), real-time RT-PCR-based SARS-CoV-2 RNA detection (TaqManTM 2019-nCoV Assay Kit v1 or v2 from Thermofisher) [24], RNA reverse transcription through multiplexing PCR (primer scheme nCoV-2019/V1) following the ARTIC protocol (https://artic.network/) [109], cDNA purification (AMPure XP beads, Agencourt), cDNA quantification (Qubit dsDNA HS Assay Kit and Qubit fluorometer 2.0 from Thermofisher or QuantiFluor ONE dsDNA System from Promega and FLUOstar OMEGA from BMG Labtech), NGS library preparation (Illumina DNA Prep kit) and sequencing (2 × 150 bp: MiniSeq or NextSeq500 from Illumina).

### Variant calling analysis

With the objective to avoid the time consuming MSA [47–52] and propose an accurate, discriminant and fast phylogenomic inference, the reference genome mapping [40–43], variant calling analysis [44–46] and functional annotation of variants (pan-SNPs, -MNPs and -InDels) [110, 111] were preferred to de novo assembly [25, 39] or consensus sequences [27]. More precisely, we implemented a mapping-based variant calling analysis including functional variant annotations based on Trimmomatic (version 0.36, parameters: illuminaclip:2:30:10, leading:25 trailing:25 slidingwindows:20:25, minlen: 36) [71], BWA (version 0.7.17, algorithm mem, default parameters) [42], FreeBayes (version 1.3.2, default parameters) [45] and SNPeff (version 4.3, default parameters) [110] implemented in Snippy (version 4.5.1, default parameters) [112] because this workflow is fast and already well packaged in Docker (Fig. 1). The usual SARS-CoV-2 reference genome Wuhan-Hu-1 (i.e. NC_045512) was used for read mapping and variant calling analysis.

### Identification of lineages

Faced to the rareness of other tools dedicated to lineage identification of SARS-CoV-2 (Nextstrain [113], GISAID [114] and PhenoGraph-based [115]), the workflow PANGOLIN has been implemented in the GENPAT workflow (Fig. 1) to assign PANGO lineages with a multinomial logistic regression-based machine learning coupled to a dynamic nomenclature of mutations associated with important functional evolution events [34]. More in details, consensus sequences were derived from BWA-based read mapping [42] with the program iVar (version 1.3, parameters: minimum length of read to retain after trimming m = 1, minimum quality threshold for sliding window to pass q = 20) [27] before to be used as input of the workflow PANGOLIN (version 3.1.11, algorithm pangoLEARN, default parameters) [34] (Fig. 1). In brief, this PANGO dynamic nomenclature proposes to label major lineages with a letter starting from the earliest lineage A SARS-CoV-2 viruses closely related to the most recent common ancestor (MRCA) Wuhan/WH04/2020 (EPI_ISL_406801) isolated from the Hubei province (China) on 5 January 2020. The early representative SARS-CoV-2 sample of the lineage B was isolated on 26 December 2019: Wuhan-Hu-1 (GenBank accession no. MN908947). Then, the dynamic nomenclature assigns a numerical value for each descending lineage from either lineage A or B (e.g A.1 or B.2) following roles with corresponding criteria [34].

### Phylogenomic inferences

Keeping in mind the objective to build phylogenomic trees matching the PANGO lineages, with high discriminatory power, and as fast as possible, we replaced the slow substitution model-based phylogenomic inference [53–55] by MST inferred with the algorithm “MSTree V2” implemented in GrapeTree (version 2.2, default parameters) [68] (Fig. 1). Similarly to cg/wgMLST workflows which use alleles of homologous genes to build MST, we propose in the present manuscript an algorithm called “vcf2mst.pl” to infer MST from functional annotation of variants (Fig. 1). This algorithm “vcf2mst.pl” (version 1.0, default parameters) uses sample dependent VCF files from upstream reference genome based-variant calling analysis (Fig. 1) to build a binary matrix of genotypes representing unique functional annotations of variants encoded in these VCF files (Fig. 2). The three main steps of this algorithm “vcf2mst.pl” aims to (1) derive functional annotations of variants (i.e. genotypes) encoded in variant calling format (vcf) into lists of samples and genotypes (lst), (2) build a scheme of genotypes (sch) and (3) create a binary matrix of genotypes according to samples of interest (Fig. 2). This algorithm “vcf2mst.pl” encodes the unique genotypes of SNPs and MNPs according to the nucleotide pattern “reference genotype - position - alternative genotype” (e.g. snp: C241T), while the unique genotypes of InDels are encoded following the nucleotide pattern “position - reference genotype - alternative genotype” (e.g. ins:11287-G-GTCTGGTTTT or del:11287-GTCTGGTTTT-G). In contrast, the unique genotypes from GISAID (i.e. ZAPPO_GISAID_VCF) are encodes following the amino acid patterns “gene name _ reference amino acid _ position _ alternative amino acid” for SNPs and MNPs (e.g. NSP12_P323L or Spike_D614G), “gene name _ ins _ position _ amino acid” for insertions (e.g. NSP6_ins35VL) and “gene name _ amino acid _ position _ del” for deletions (e.g. NSP1_M85del). The proposed MST-based phylogenomic inference is able to manage together pan-SNPs, -MNPs and -InDels (i.e. core and accessory variants) with respect to the reference genome, because the presence of alternative genotype is encodes “1”, while the absence of alternative genotype is encodes “0”.

## Supplementary Information

**Additional file 1. **Metadata and PANGO lineages of the dataset of SARS-CoV-2 samples isolated by IZSAM in provinces of the Abruzzo region (Italy), then shotgun sequenced and analyzed in GENPAT until April 2021 (*n* = 1592).

**Additional file 2. **Metadata and PANGO lineages of the dataset of SARS-CoV-2 samples isolated from several Italian provinces and retrieved by GENPAT from GISAID until April 2021 (*n* = 17,201).

**Additional file 3. **Distributions per Italian provinces (*n* = 25) of the SARS-CoV-2 PANGO lineages in Italy (*n* = 176) retrieved by GENPAT from GISAID until April 2021 (*n* = 17,201).

**Additional file 4. **Newick file inferred through variant calling-based analysis, binary matrix of functional annotations of variants (pan-SNPs, -MNPs and -InDels) with the program “vcf2mst.pl” and Hamming-like distance-based minimum spanning tree (MST) implemented in GrapeTree (“MSTree V2”), from the dataset of SARS-CoV-2 samples isolated by IZSAM in provinces of the Abruzzo region (Italy), then shotgun sequenced and analyzed in GENPAT until April 2021 (*n* = 1553).

**Additional file 5. **Newick file inferred through variant calling-based analysis, binary matrix of functional annotations of variants (pan-SNPs, -MNPs and -InDels) with the program “vcf2mst.pl” and Hamming-like distance-based minimum spanning tree (MST) implemented in GrapeTree (“MSTree V2”), from the dataset of SARS-CoV-2 samples isolated from several Italian provinces and retrieved by GENPAT from GISAID until April 2021 (*n* = 15,451).

## Data Availability

Metadata and consensus sequences of all the SARS-CoV-2 are available from GISAID (https://www.gisaid.org/) at the accession numbers described in supplementary information. The algorithm “vcf2mst.pl” is available in GitHub (https://github.com/genpat-it/vcf2mst).

## Abbreviations

ARDS: Acute respiratory distress syndrome
cg/wgMLST: Coregenome and whole genome multi-locus sequence typing
CoV: Coronavirus
COVID-19: Coronavirus disease 19
GATK4: Genomic analysis toolkit
GENPAT: Whole Genome Sequencing of microbial pathogens: data-base and bioinformatics analysis
GISAID: Global Initiative on Sharing All Influenza Data
IZSAM: Istituto Zooprofilattico Sperimentale dell’Abruzzo e del Molise Giuseppe Caporale
ML: Maximum likelihood
MSA: Multiple sequence alignment
MST: Minimum spanning trees
NGS: Next generation sequencing
NRC: National Reference Centre
RdRp: RNA-dependent RNA polymerases
SARSr-CoVs: SARS-related coronaviruses
SARS-rCoV: Severe acute respiratory syndrome-related virus
ST: Sequence type
UK: United Kingdom
VCF: Variant calling format
WHO: World Health Organization
